# Dynamic changes in tooth displacement and bone morphometry induced by orthodontic force

**DOI:** 10.1038/s41598-022-17412-8

**Published:** 2022-08-11

**Authors:** Chen Zong, Jeroen Van Dessel, Greetje Vande Velde, Guy Willems, Maria Cadenas de Llano-Pérula

**Affiliations:** 1 Department of Oral Health Sciences-Orthodontics, KU Leuven and Dentistry, University Hospitals Leuven, Kapucijnenvoer 7, blok A, bus 7001, 3000 Leuven, Belgium; 2 Oral and Maxillofacial Surgery, University Hospitals Leuven and OMFS-IMPATH Research Group, Department of Imaging & Pathology, Faculty of Medicine, KU Leuven, Leuven, Belgium; 3 Biomedical MRI/Molecular Small Animal Imaging Center (MoSAIC), Department of Imaging & Pathology, Faculty of Medicine, KU Leuven, Leuven, Belgium

**Keywords:** Dental radiology, Occlusion, Orthodontics, Dentistry, Resorption

## Abstract

This study used a novel 3D analysis to longitudinally evaluate orthodontic tooth movement (OTM) and bone morphometry. Twelve-week-old male Wistar rats were subjected to OTM by applying a constant orthodontic force (OF) of 25cN between one of the upper first molars and a mini-screw. In vivo micro-CTs were taken before and after 10, 17, 24 and 31 days of force application, and superimposed by a novel and rigid voxel-based registration method. Then the tooth and alveolar bone segment at different time points became comparable in the same coordinate system, which facilitated the analysis of their dynamic changes in 3D. By comparison between time points and between OF and no OF sides, this study showed that the OTM rate was not constant through time, but conformed to a ‘V’ shape changing pattern. Besides, OF induced displacement of both loaded and unloaded teeth, and the latter mirrored the former in a delayed manner. In addition, bone morphometric changes synchronized with OTM rate changes, implying that a higher OTM rate was concomitant with more alveolar bone loss. The pressure and tension areas might not be in two opposite sides, but actually adjacent and connected. These findings might provide instructive evidence for both clinical, translational and basic research in orthodontics.

## Introduction

Shortening orthodontic treatment time is of interest to both clinicians and patients^1^. As a result, numerous techniques and products have been commercialized in the last years aiming to accelerate orthodontic tooth movement (OTM)^2,3^. However, due to the high methodological heterogeneity of OTM evaluation, there is still a lack of knowledge regarding the physiology of tooth movement^4,5^. Basic research in orthodontics often focuses on a microscopic level, such as on the role of specific molecules, signaling pathways or gene expression patterns involved in OTM^6–10^. However, macroscopic changes induced by orthodontic force are less studied, despite being highly clinically relevant. This can partially be due to the difficulties inherent to this research model: studies in human would involve biopsies or increased radiological exposure only for research purposes, which cannot be justified. Orthodontic animal models, mostly developed in rodents, offer important opportunities. However, research derived from them often presents contradictory results, probably due to high methodological heterogeneity, such as the use of different orthodontic anchorage, forces and appliances.

Classically, OTM has been divided into different phases according to the rate of tooth displacement. The initial phase would be followed by an arrest period (lag phase), after which an acceleration of OTM would occur, followed by a linear increase in tooth displacement^11–13^. However, studies usually measure this displacement as a distance, which cannot reflect the real 3D situation, since displacement is actually a vector. Second, the dynamic change in alveolar bone when applying a constant orthodontic force remains largely unknown. Although previous animal research showed changes in the bone mineral density (BMD) of alveolar bone subjected to orthodontic force^14,15^, few studies performed complete longitudinal bone morphometric assessment^16,17^. Whether there is a correlation between the dynamic changes in OTM rate and the changes in bone morphometric parameters still needs to be elucidated.

The aim of this study is to investigate the dynamic changes induced by orthodontic force in tooth displacement and bone morphometry in a rat OTM model over 31 days by using a novel 3D analysis and bone anchorage. These dynamic changes can provide important evidence regarding the optimal window periods to investigate microscopic molecular changes. In addition, the potential correlation between OTM rate and bone morphometry at different time points after the application of orthodontic force could directly provide instructive guidelines not only for basic research, but also for the clinicians.

## Materials and methods

### Animals

Twelve young adult male Wistar rats (9 weeks old) were used in this study. Weekly surveillance was performed in order to notice any changes in weight and guarantee normal feeding. Prior to the experiment, the minimum sample size was calculated using a previous split-mouth study^18,19^. The primary parameter considered was the mesial displacement of the first molar after 31 days of orthodontic force application. A power analysis in the software G*Power 3.1 (Düsseldorf, Germany) suggested a minimum sample size of 12 animals per group (OF and no OF side) for a repeated measures MANOVA when assuming 99% power with α = 0.05.

The animals were housed in three cages under continuous temperature (23 °C), a 12-h schedule alternating light and dark, and standard rat maintenance diet. All animal experiments were performed in the Laboratory Animal Center and the Molecular Small Animal Imaging Center (MoSAIC) of KU Leuven, Belgium, with the approval of KU Leuven Ethical Committee for Animal Experimentation (P197/2019) and in accordance with the EU Directive 2010/63/EU and ARRIVE 2.0. Guidelines.

### Interventions

**Figure 1:**
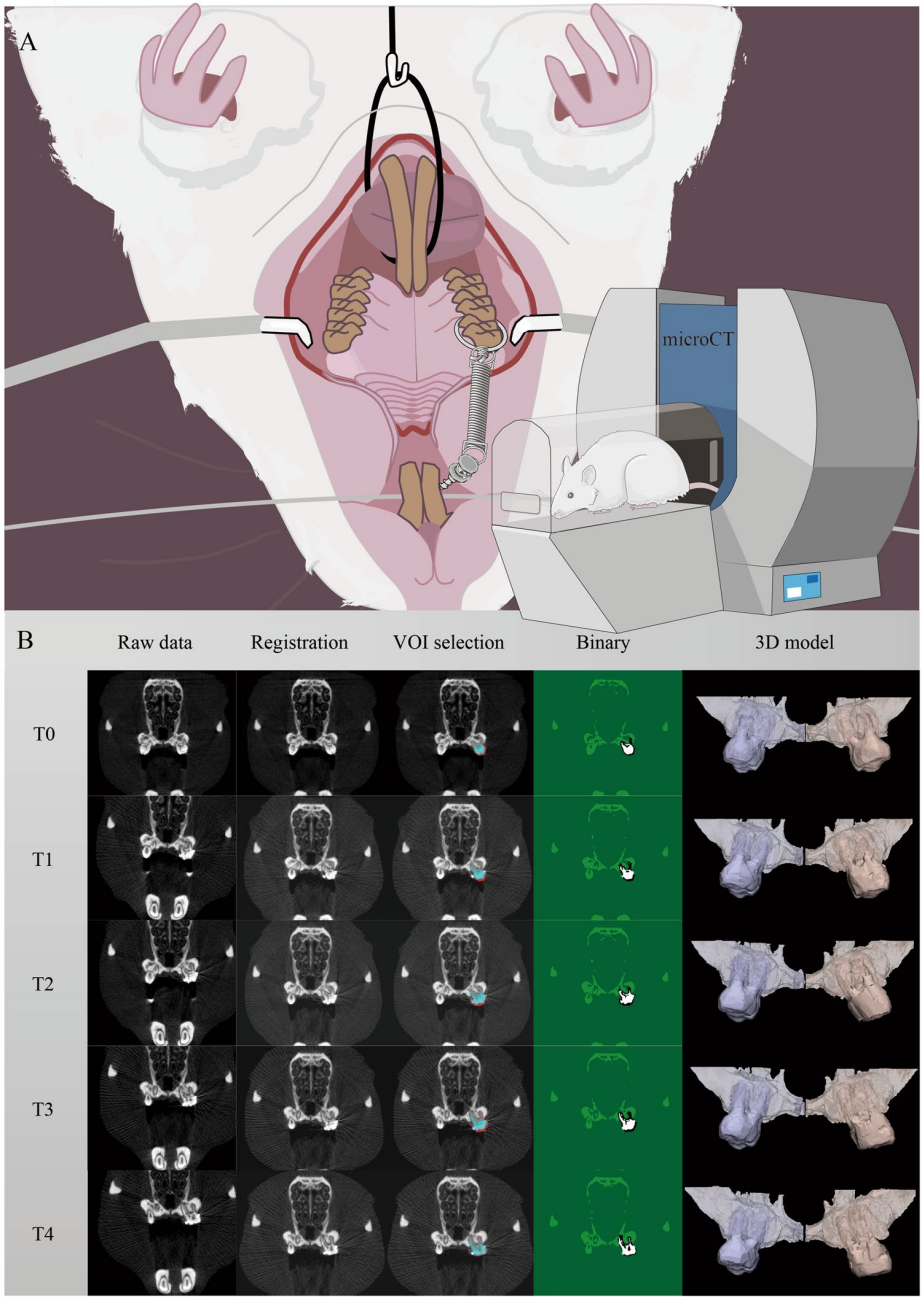
OTM Animal model. (**A**) Graphic representation of the animal model. A constant force of 25 cN was loaded between by a spring coil between the first molar and mini-screw in the OF side. The rats were scanned using a low-dose in vivo micro-CT at five different time points (T0–T4). (**B**) Overview of the image processing workflow. Micro-CT scans from different time points (T1–T4) were spatially aligned with the micro-CT on baseline (T0). A volume of interest (VOI) was delineated on the reconstructed micro-CT data to ensure an accurate selection of teeth and alveolar bone. These were segmented using a global threshold algorithm. Based on the resulting binary images an automatic 3D morphometric quantification was performed and 3D models were created.

Tooth movement was performed by following a previously published protocol^18^. Briefly, on one randomly-allocated side of the maxilla, a self-drilling mini-screw (2.5 mm × 1.3 mm × 5 mm, DEWIMED, Tuttlingen, Germany) was implanted in the alveolar bone, approximately 2 mm distal to the upper incisors and with an angulation of 45° to the occlusal plane, in order to provide bone anchorage. Healing time of 3 weeks was allowed to ensure stability^20^. Afterwards, a constant orthodontic force (OF) of 25 cN was loaded between the mini-screw and the upper first molar by using a Sentalloy closed coil spring (Ultra-light, Dentsply GAC, Rochecorbon, France) (Fig. 1A). At the contralateral side, no OF was applied (no OF side). At this moment, the animals were 12 weeks old.

All interventions were conducted by the same investigator, firstly under sedation of 2.5–5% isoflurane (1000 mg/g, Iso-Vet, Dechra, Skipton, UK), followed by anesthesia of 100 mg/ml IP ketamine (80 mg/kg, Nimatek, Bladel, Netherlands) and 2% xylazine (10 mg/kg, XYL-M, V.M.D, Arendonk, Belgium). After the interventions, soft diet and analgesic medication (0.05 mg/kg Buprenorphine) were supplied for 3 days. To prevent unnecessary animal suffering and potential appliance loss, daily surveillance of body weight and intraoral examination under sedation with isoflurane was performed. The rat grimace scale^21^ was used to evaluate whether animals were in pain. A schematic diagram showing treatments of the rats from arrival to euthanasia is shown in Supplementary Fig. 1.

### Micro-CT

Animals were longitudinally followed up with micro-CT at five time points: right before the application of OF (T0, baseline), and 10 (T1), 17 (T2), 24 (T3), and 31 (T4) days after OF. A low-dose, high-resolution in vivo micro-CT (Skyscan 1278, Bruker, Kontich, Belgium) was used for image acquisition. A high-resolution scan protocol was used at 65 kVp, 500 μA, and 180° with an angular rotation step of 0.5°, resulting in an exposure time of 50 ms. A 1 mm aluminum filter was used to eliminate the beam hardening effect. Flat field correction was performed for calibration based on an empty field of view previous to the actual scans. Animals were placed under sedation of 2.5–5% isoflurane during image acquisition. After image acquisition, the image stacks were reconstructed with NRecon software (version 1.7.1, Bruker, Kontich, Belgium). Correction for post-alignment, and ring-artifacts reduction were optimized per scan if needed. Smoothing level and beam hardening were applied with values of 0 and 10%, respectively. The dynamic image range of histogram was set from 0.003 to 0.03.

### Longitudinal assessment of OTM

The dynamic changes of OTM were volumetrically evaluated based on the spatial displacement of the teeth from T0 to T4, using T0 as a baseline without depending on other external reference structures. Rigid voxel-based registration steps were performed before the evaluation to ensure that all teeth were in the same coordinate system and were temporospatially comparable at each time point.

First, the follow-up micro-CT scans at T1, T2, T3 and T4 were manually superimposed with the corresponding baseline scan at T0 based on the maxillary structures as a reference, followed by an optimized automatic superimposition by MTM Scaffold Strain (KU Leuven, Leuven, Belgium)^22,23^. The structural compatibility of maxillary anatomical reference points was inspected to verify the validity of the manual and automatic superimposition. Second, the maxilla, mini-screw, first and second molars at each time point were delineated by the same investigator and saved as volumes of interest (VOI) in CTAnalyser software (version 1.17.5, Bruker, Kontich, Belgium). Third, the selected VOIs were automatically segmented using an adaptive threshold algorithm^22,23^ and imported as individual 3D standard tessellation language (stl) models, which were loaded in 3-Matic (Materialise, Leuven, Belgium) for longitudinal assessment of OTM and bone morphometry. The complete workflow is shown in Fig. 1B.

**Figure 2:**
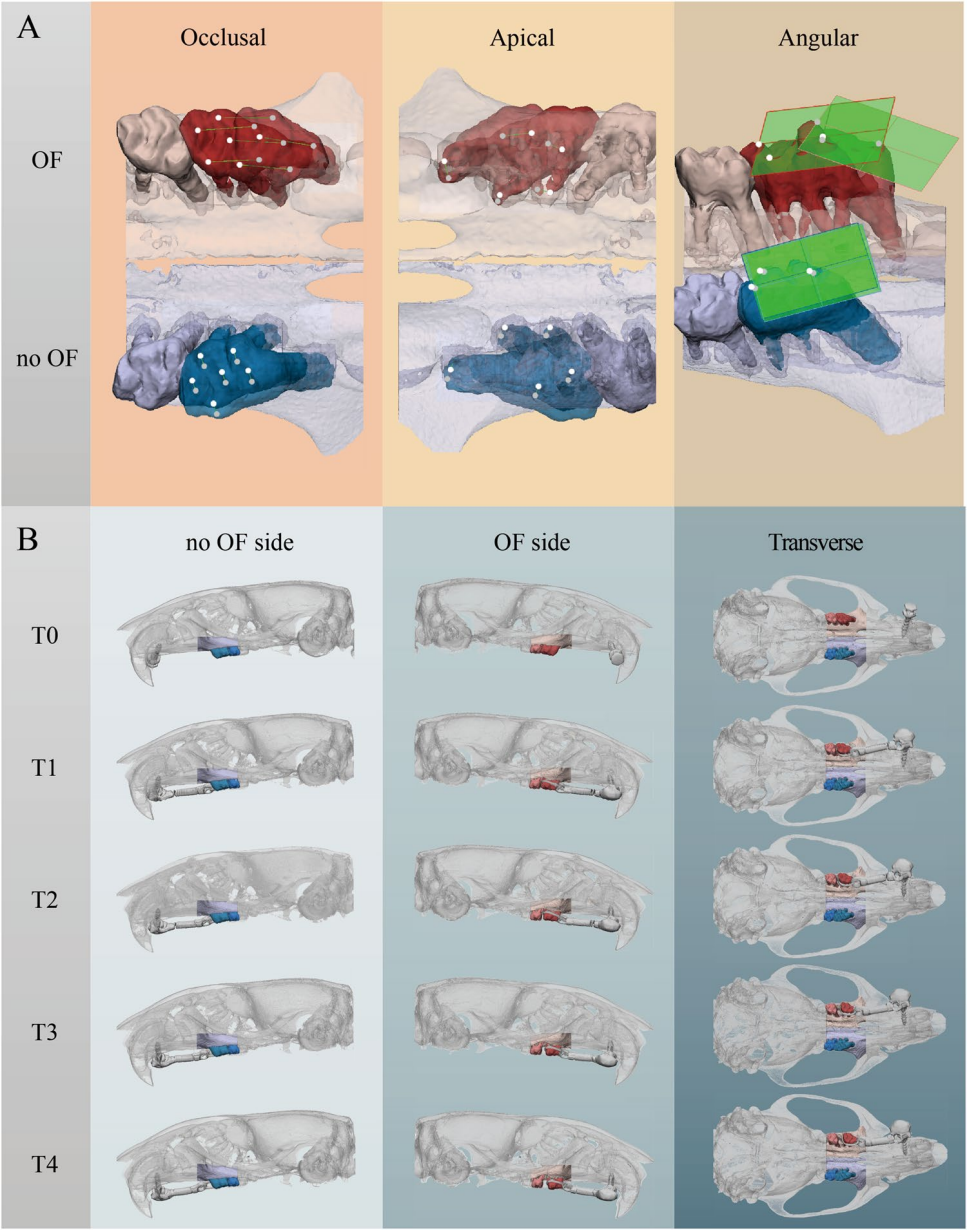
Longitudinal assessment of OTM. (**A**) In the T0 model, occlusal, apical points and occlusal plane were defined. Then, the same points were extrapolated into the T1–T4 models by automated global registration. The distances or angles between them were then measured as occlusal, apical or angular movement. (**B**) Dynamic changes in molar displacement and alveolar bone morphometry. (Red = OF side, Blue = No OF side).

To assess first and second molar displacement, six reference points on the cusps and five on the root apices were created. Their displacement over the different time points was defined as occlusal and apical movement, respectively. The occlusal plane was defined by the mesial cusp, distobuccal and distolingual points. The angle between the occlusal planes was defined as angular movement. The occlusal, apical and angular movements of the first and second molars at T0–T1, T0–T2, T0–T3, T0–T4, T1–T2, T2–T3, T3–T4 were measured on both OF and no OF side. The OTM rate was calculated by the occlusal movement divided by the time of OTM. The longitudinal measurements of the above-mentioned parameters and their dynamic changes are shown in Fig. 2.

For assessment of mini-screw displacement, two reference points were created on the center of the screw head and the screw tip in the T0 model. The line connecting these two points was defined as the central axis. Then, the distances of the head and tip points from T0 to T1 were measured as the head and tip movement in the T0–T1 period, respectively. The angle between the central axes in T0 and T1 was measured as the angular movement. In this way, the displacement of mini-screw at T0–T2, T0–T3, T0–T4, T1–T2, T2–T3, T3–T4 was also measured.

### Longitudinal assessment of bone morphometry

**Figure 3:**
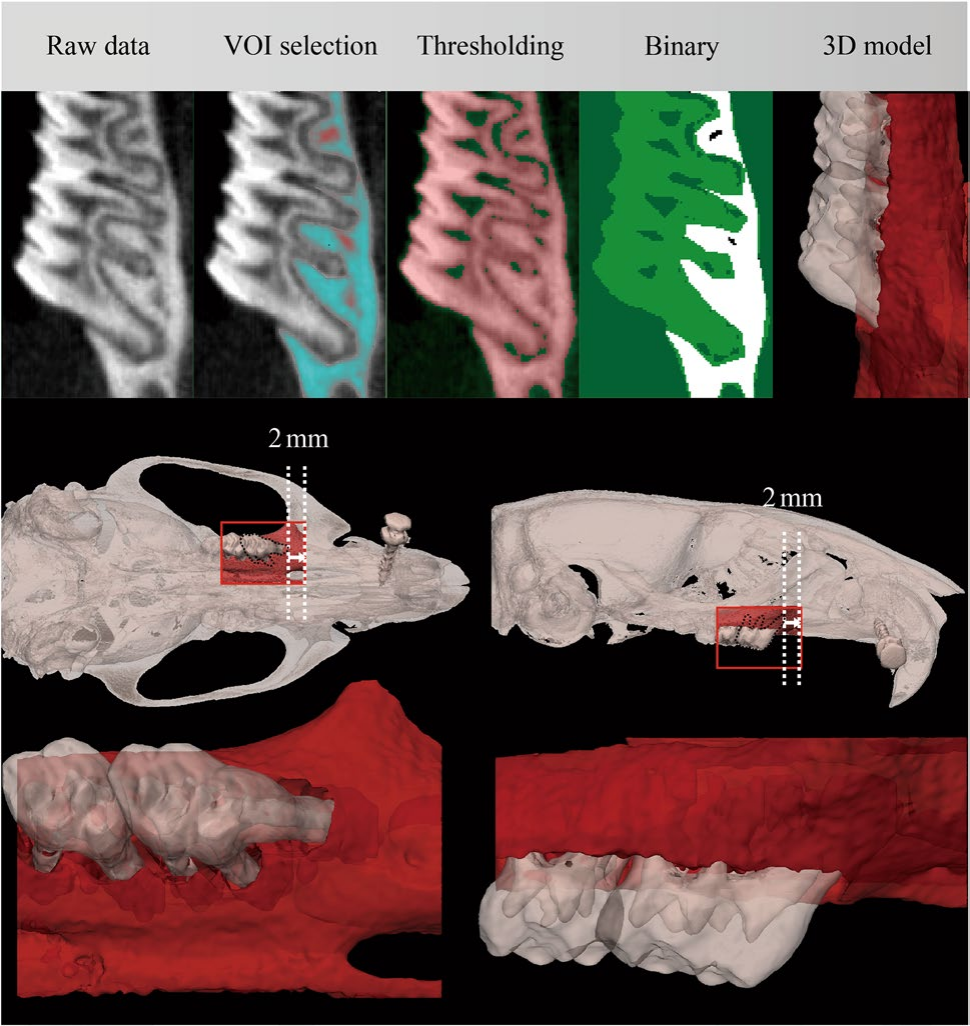
Longitudinal assessment of bone morphometry. The VOIs of the alveolar bone, first and second molar were separately selected and segmented as 3D models on the registered micro-CT in a workflow. The bone morphometry was evaluated in VOIs at each time points and compared longitudinally.

Alveolar bone morphometry was quantified using the method proposed by Chatterjee et al., as shown in Fig. 3^24^. The registered micro-CT images were transferred to CTAnalyser and a volume of interest (VOI) containing only the molar region of each hemi maxilla was selected by defining a top and a bottom slice. The top slice was defined as the slice 2 mm mesial from that where the cusp of the first molar appeared. The bottom slice was defined as that where the mesial cusp of the third molar appeared. The alveolar bone within this region was semi-automatically selected and segmented using an automatic global threshold algorithm.

To evaluate the BMD, phantoms (standard hydroxyapatite blocks) of 0.25 g/cm^3^ and 0.75 g/cm^3^ were scanned to perform BMD calibration with respect to the attenuation values. The BMD at the different time points was calculated by linear extrapolation using: Y $-{y}_{1}= ({y}_{2}-{y}_{1})/({x}_{2}-{x}_{1}) \times$ (X $-{x}_{1}$), where x_1_ and x_2_ are the greyscale indices of standard hydroxyapatite 0.25 and 0.75 g/cm^3^, y_1_ and y_2_ are known as 0.25 and 0.75 g/cm^3^, and X and Y are the grey indices.

The dynamic changes of the following parameters were also evaluated in VOIs for the assessment of bone morphometry in CTAnalyser:

1. Bone volume fraction (BV/TV, %): the proportion of the VOI occupied by binarised solid objects in 3D within the VOI, which directly reflects the bone volume.
2. Bone surface density (BS/TV, mm^−1^): the ratio of surface area to total volume in 3D within the VOI.
3. Trabecular number (Tb.N, mm^−1^): the number of traversals across a trabecular or solid structure made per unit length on a random linear path through the VOI.
4. Trabecular thickness (Tb.Th, mm): essentially the thickness of the solid voxels as defined by binarisation within the VOI.
5. Trabecular separation (Tb.Sp, mm): essentially the thickness of the spaces among trabecular bone.

### Statistical analysis

Two-way repeated measures MANOVA and post-hoc Tukey’s test were used to compare the OTM and bone morphometry between different time points (T0–T4) and between the OF and no OF side. Non-parametric analysis (Friedman test) was performed to compare the displacement of the mini-screw among different time points. The correlation of the dynamic changes between bone morphometric parameters and OTM rate was evaluated by Pearson correlation coefficients. Non-parametric statistical methods were used when normality was not confirmed using the Kolmogorov–Smirnov test. Statistical analysis was performed in GraphPad (version 8.4.3, San Diego, USA).

## Results

No relevant weight loss was detected in the animals throughout the experiments.

### Dynamic changes in OTM

**Figure 4:**
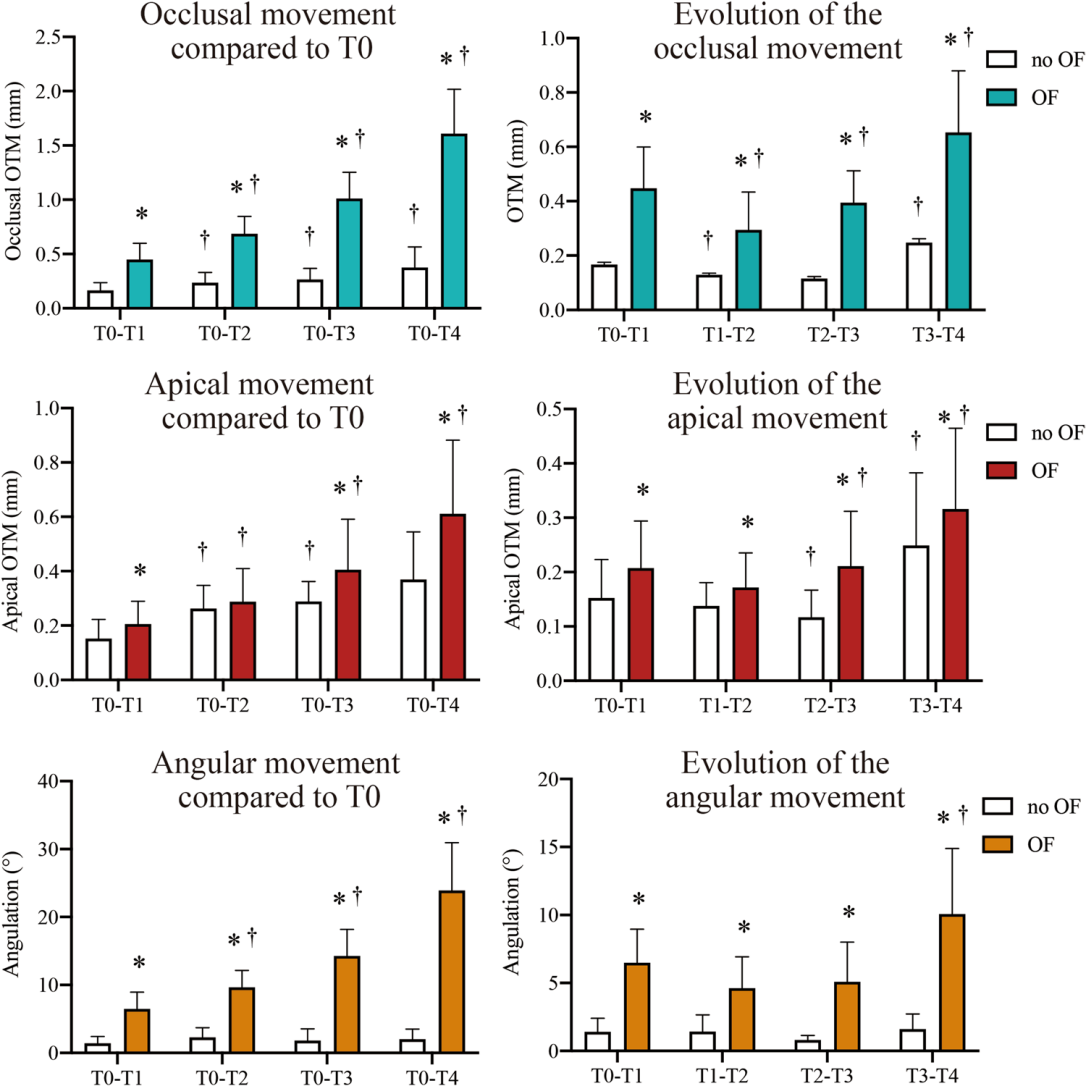
First molar displacement. The OTM was compared between different time points (T0–T4) and between the OF and no OF side by two-way repeated measures MANOVA and post-hoc Tukey’s test (n = 12). *P < 0.01 versus no OF side. †*P* < 0.01 versus previous period.

**Figure 5:**
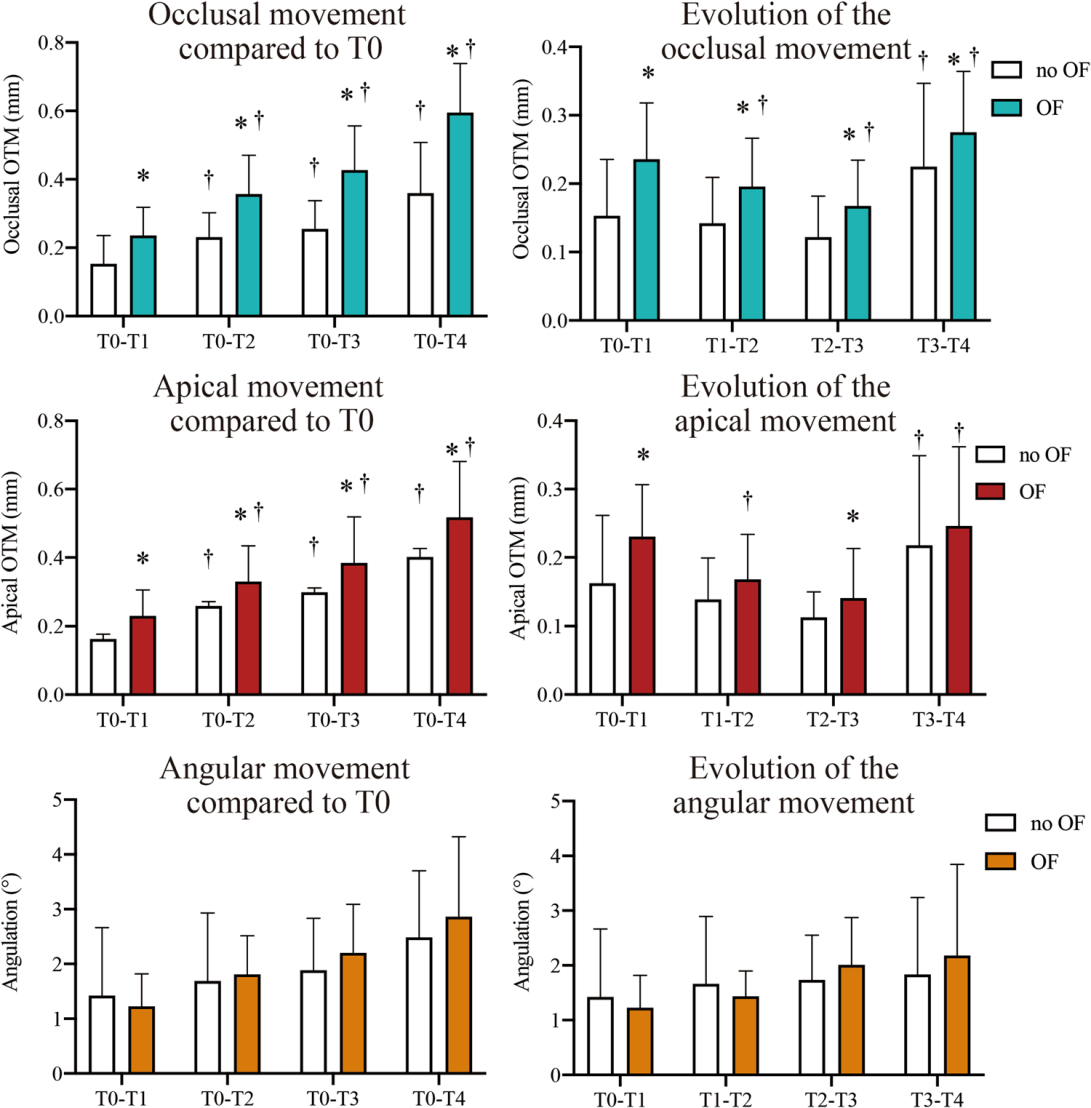
Second molar displacement. The OTM was compared between different time points (T0–T4) and between the OF and no OF side by two-way repeated measures MANOVA and post-hoc Tukey’s test (n = 12). *P < 0.01 versus no OF side. ^†^*P* < 0.01 versus previous period.

Significant differences in occlusal, apical and angular movement were found among the different time points (*P* $<$ 0.01) for both the first (Fig. 4) and second molars (Fig. 5) in the OF side. The OTM rate of the first molar was not constant among the different time points, but conformed to a ’V’ shape changing pattern: it first decreased from T1 to T2 (*P* $<$ 0.01), and then increased from T2 to T4 (*P* $<$ 0.01).

Remarkably, the first and second molars of the no OF side also underwent significant displacement (*P* $<$ 0.01) after 31 days of force application on the contralateral hemi-maxilla. Although of course significantly less OTM was found compared to the OF side (*P* $<$ 0.01), the changing pattern of OTM rate of the second molar at the OF side and of both molars at the no OF side was similar to that of the loaded first molar.

### Displacement of the mini-screw

**Figure 6:**
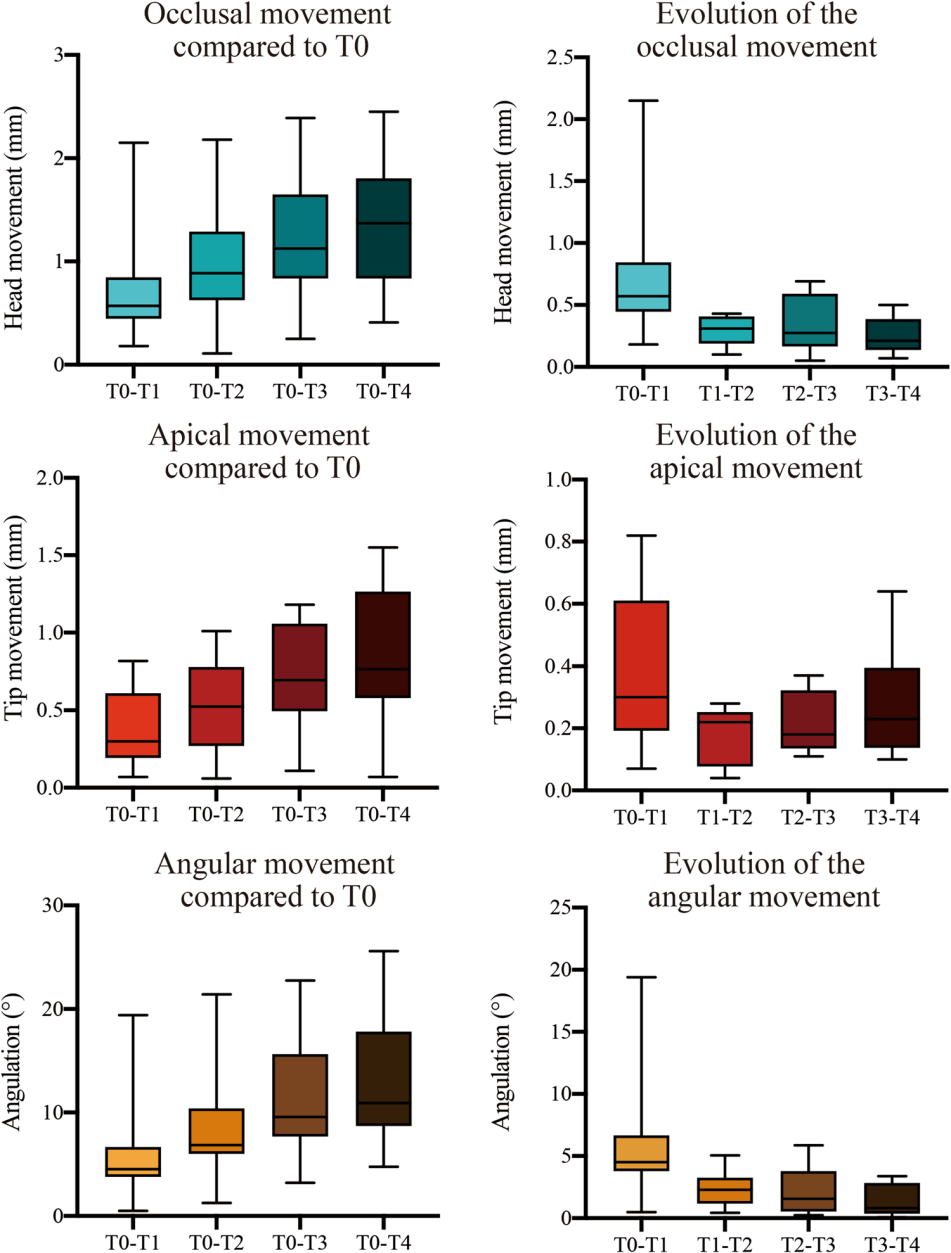
Mini-screw displacement. The displacement of the mini-screw was compared among different time points by Friedman test (n = 12). *P < 0.05.

No mini-screw loosened or was fully lost during the follow-up period. However, mini-screw displacement was observed, shown by box plots (minimum, Q1, median, Q3, maximum) in Fig. 6. The non-parametric analysis (Friedman test) shows the mini-screw had a significantly different displacement on the head (*P* $<$ 0.01) but not on the tip (*P* $>$ 0.05) over time.

### Dynamic changes in bone morphometry

**Figure 7:**
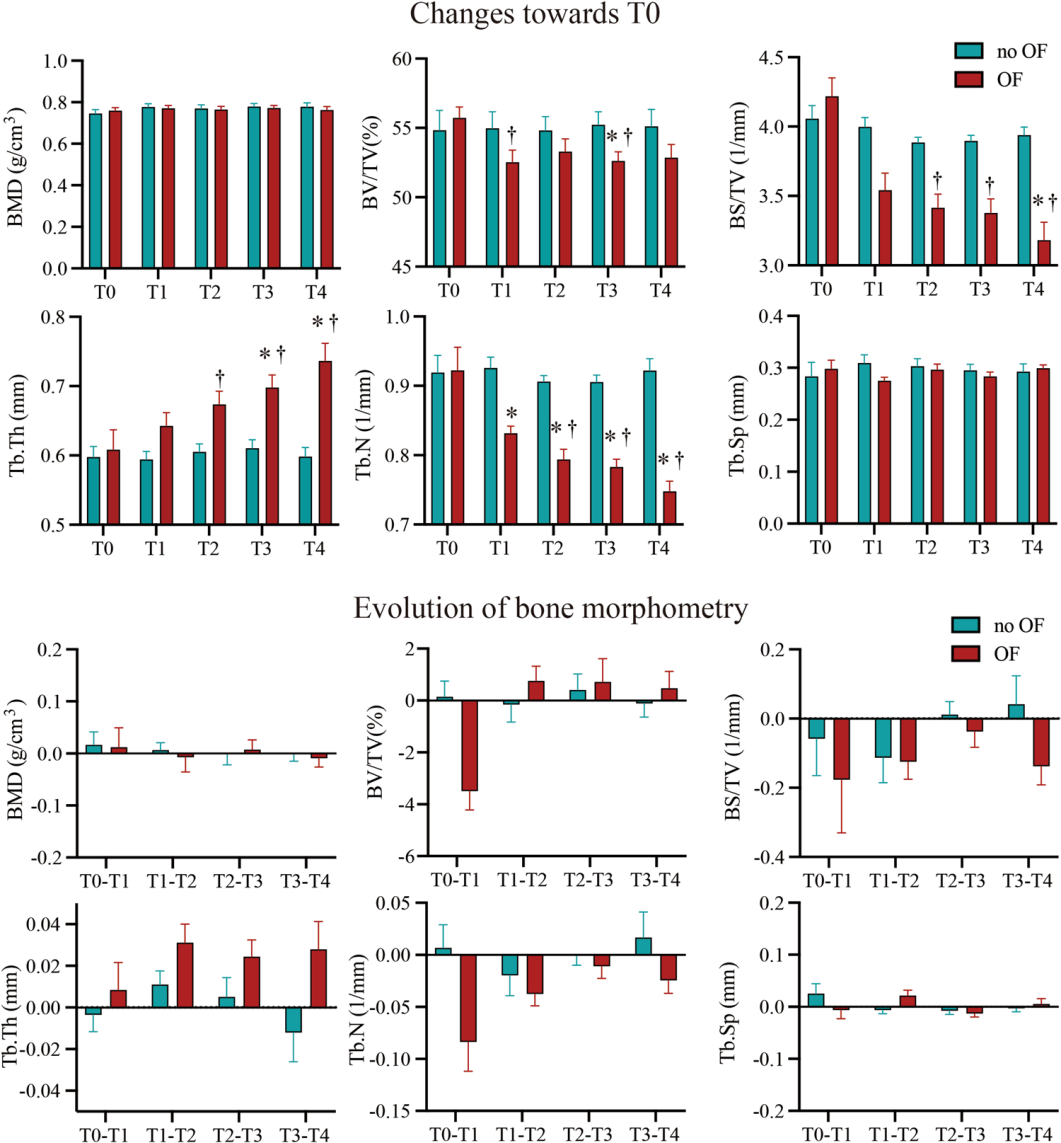
Dynamic changes in bone morphometry. The bone morphometric parameters were compared between different time points (T0–T4) and between the OF and no OF side by two-way repeated measures MANOVA and post-hoc Tukey’s test (n = 12). BMD = bone mineral density, BV/TV = bone volume fraction, BS/TV = bone surface density, Tb.Th = trabecular thickness, Tb.N = trabecular number, Tb.Sp = trabecular separation. *P < 0.01 versus no OF side. ^†^*P* < 0.01 versus previous period.

Alveolar bone morphometry was quantified and compared between the OF and no OF sides and among different time points (T0–T4) by two-way repeated measures ANOVA. The dynamic changes in bone morphometry are shown in Fig. 7.

When comparing the OF and no OF sides, no significant difference in bone mineral density (BMD, g cm^−3^) was observed (*P* $>$ 0.05). However, bone volume fraction, bone surface density and trabecular number (BV/TV, %, BS/TV, mm^−1^ and Tb.N, mm^−1^) decreased significantly in the OF side compared to the no OF side (*P* $<$ 0.05). In contrast, trabecular thickness (Tb.Th, mm) increased significantly (*P* $<$ 0.05). Trabecular separation (Tb.Sp, mm) had no significant difference between both sides (*P* $>$ 0.05).

**Figure 8:**
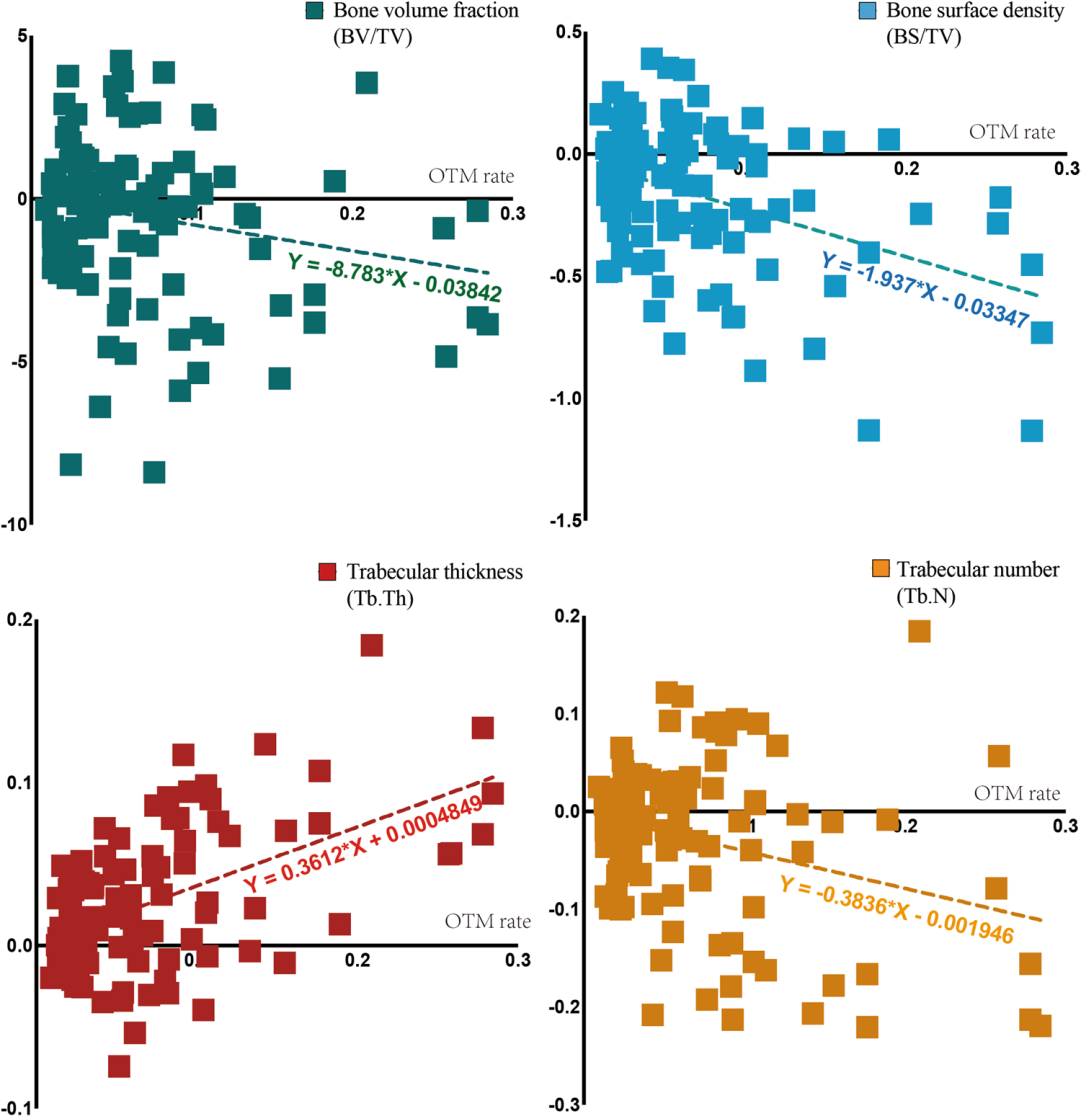
Agreement between the changes in OTM rate and bone morphometry. The OTM rate negatively correlated with bone volume and trabecular number, and positively correlated with trabecular thickness (n = 12, *P* $<$ 0.05). Besides, there is no significant linear correlation between the OTM rate and BMD or trabecular separation (*P* $>$ 0.05, not shown in this figure).

When comparing the different time points, BMD and trabecular separation had no significant change (*P* $>$ 0.05). But BS/TV and Tb.N decreased significantly (*P* $<$ 0.05) while Tb.Th increased significantly (*P* $<$ 0.01) with time. Additionally, the dynamic changes of BV/TV, BS/TV, Tb.Th and Tb.N conformed to a ‘V’ shape changing pattern, showing a linear correlation with the change of OTM rate (*P* $<$ 0.05) in Fig. 8.

## Discussion

From digital calipers to intraoral scanners, from histology to cephalograms and from 2 to 3D, the methodology to measure in vivo OTM has been developing for years^19,25–30^. Although measurements have become more and more accurate, the evaluation of the dynamic changes induced by OF still remains difficult, as it is hard to find a reliable reference structure. Previous studies in rats evaluated 2D or 3D OTM in an indirect way based on external reference structures such as the contralateral maxilla (split-mouth designs)^19,31^, the incisors^28,32^, the zygomatic arch^17^ or the diastema formed between the first and the second molar^10,15,33–36^. However, these methods rely on those reference structures remaining stable during OTM. The present study uses a novel method to longitudinally evaluate dynamic OTM regardless of external reference structures^18^. After rigid voxel-registration steps based on the maxilla, the scans at different time points become comparable throughout time in the same coordinate system. Thus, the tooth becomes its own self-control for OTM assessment.

Another novelty of this study lies on the dynamic measurements. First, OTM was truly evaluated in 3D instead of 2D or pseudo 3D (using a reconstructed cephalogram from CT). This is a clear advantage since real OTM is a compound displacement of vertical, transversal and sagittal movement. Second, occlusal, apical and angular movement were evaluated separately, instead of performing an average, since not only the compound body movement but also tipping and torque are crucial to orthodontists. Third, OTM was calculated both relative to T0 and between successive time points, in order to register OTM evolution. Since the true 3D displacement of the same tooth did not necessarily always happen in the same direction or plane (Supplementary Fig. 2), the evolution of OTM should be evaluated not simply by subtracting the OTM towards T0 of the adjacent periods (for example T0–T1 and T0–T2), but by directly measuring the OTM between each adjacent period (T1–T2). This represents a step forward from previous cephalometric, geometric and optometric measurements.

In the present study, 3D analysis of first molar displacement allowed us to study its pattern. After an initial phase of movement, a marked decrease in OTM rate was noted 10 days after the application of force, followed by linear increase 1 week later. This matches the reports of classical articles regarding the phases of tooth movement^11–13^. Since the observation time of our study was 31 days and most related articles only include observation times up to 4 weeks, no statements can be made regarding the possible changes happening beyond this point.

The most surprising finding of our study regards the displacement of unloaded teeth. The second molar was found to significantly displace in a mesial direction in the OF side, although no OF was directly applied on it. This phenomenon confirms the unreliability of choosing the diastema formed between first and second molars as a reference point for OTM assessment. Similarly, significant movement of the first and second molars in the no OF side was also found, following the same pattern described in the OF side. However, this pattern is in contradiction to that reported by previous studies in which the OTM in the no OF side was considered to be zero^37^. A possible explanation is that the 2D measurements used in these studies may have missed displacement happening in planes other than sagittal.

Considering the fact that rat molars migrate distally during aging^38,39^, the tooth mesialization in the no OF side found in the present study cannot be due to physiologic movement. It could be an adaptive response to the change in dental occlusion and microscopic biologic environment caused by OF in the contralateral side, which is supported by the fact that the OTM in the no OF side mirrors the pattern of that in the OF side^18^ (Fig. 4, 5). The pattern of OTM of the second molar in the OF side also mirrored that of the loaded first molar but started with a short delay, probably due to the time consumed for occlusal change or the stretching from the periodontal fibers. Altogether, these findings show that the OF applied in one tooth can indeed influence the other teeth, even on the contralateral side, which can therefore no longer be regarded as “control” since tooth displacement can be camouflaged, leading to systematic underestimation of real OTM.

Besides tooth displacement, alveolar bone remodeling is crucial to clinicians. Although BMD has been previously studied and can reflect bone changes^15,40,41^, this does not allow for differential analysis of bone volume and mineralization density^42^, which together with bone volume fraction, bone surface density, trabecular thickness, number and separation are important aspects of bone morphometry, as they can reflect alveolar bone remodeling^23,43^. In the present study, significantly less bone volume (BV/TV, BS/TV) was found in the OF side compared to the no OF side, but, relevantly, there was no significant difference in BMD between sides. This implies that the bone resorption caused by the aseptic inflammation of OTM^44^ does coexist with bone formation, maintaining the average BMD. This is in contrast with what happens in pathological processes such as osteoporosis^45^ or periodontitis^46–48^, where both bone volume and BMD decrease. In correspondence to the changes in bone volume, the trabecular number was also lower in the teeth/bone on the OF side, which also presented with higher trabecular thickness. Longitudinally, both bone volume and trabecular number decreased from T0 to T4, with a constant increase in trabecular thickness as a compensation. Similarly to BMD, the trabecular separation was not significantly different between sides. Although the changes of bone morphometry compared to baseline were statistically significant, the evolution of these changes was not, probably due to the sample size of this study. To the best of our knowledge, no previous study had longitudinally assessed bone morphometry after OTM based on such a rigid voxel-based registration method. Due to this, we calculated the sample size from previous research, based on OTM instead of on bone morphometric parameters.

Longitudinal follow-up of the sample shows that the differences between OF and no OF sides are not due to accidental errors, since they were measured and visualized at each time point, and the difference was constant. Interestingly, the curves representing the changes in bone morphometry over time show a trend (Fig. 7), contrary to the ‘V’ shape changing pattern of OTM rate (Fig. 4). Linear regression analysis showed a significant correlation between the changes in OTM and BV/TV, BS/TV, Tb.N (positively) and Tb.Th (negatively). Although the correlation does not imply causation, this finding suggests that higher OTM rate was concomitant with more alveolar bone loss, which is clinically very relevant. Finding a balance between accelerating OTM and minimum alveolar bone resorption is crucial for future research and clinical practice.

It should be noted that the method of analysis is crucial to the validity of the conclusion, since results of bone morphometry depend on the VOI. Previous studies selected VOIs either in function of the pressure/tension sides^16^ or anatomic sites such as at the furcation^49,50^ or mesial root^15,51^ of the first molar, in different jaws and animal species^52,53^. However, these areas are difficult to be determined accurately. Furthermore, since OTM is not only a simple mesial body movement (also angular movement, and not necessarily only in the sagittal direction), the pressure and tension areas are not simply the mesial and distal root areas (Supplementary Fig. 3). In the present study, the whole alveolar bone around the first and second molar was selected as VOI, which should simultaneously include pressure and tension sites, since choosing only one of the two might lead to biased BMD results. On the other hand, the relatively wide VOI might reduce the statistical power, because alveolar bone not influenced by the movement might also be selected and therefore hide the change in affected bone. However, this could be compensated by the longitudinal design of the study, since the ‘non-affected’ alveolar bone is eliminated during the subtraction of the different time points.

As mentioned before, the pattern of movement of the first molar in the OF side and all the other molars was found to be similar, but delayed. It could be argued that the displacement of unloaded molars was an adaptive result to that of the loaded first molar. Since the bone morphometric changes were synchronized with the changes in OTM rate, the changes in alveolar bone could not be the cause neither the result, but concomitant with OTM. According to the classical pressure-tension theory, the mesial body movement of the tooth results from mesial alveolar bone resorption and distal bone apposition. In this case however, there should also be a delay between the critical point in the changing pattern of OTM rate and bone morphometry, which was not observed in our sample. This reinforces the idea, already presented in literature^54^, that the pressure and tension areas are not in two opposite sides, but actually dispersed, adjacent and widely connected in both mesial and distal sides (Supplementary Fig. 3), with bone resorption and formation happening simultaneously. If this is correct, maybe a tilting movement, rather than bodily displacement should be the goal in clinical orthodontics, since it could yield more efficient OTM. This discrete tilting, involving minimal tipping, could be induced by forces lower than the ones currently used clinically. Future research should focus on how to take the best advantage of the tension and pressure areas to optimize total tooth movement, taking into consideration factors such as the force magnitude or root resorption. The longitudinal 3D analysis presented in the present study can offer methodological support to future research.

## Conclusion

This study longitudinally assesses OTM and bone morphometry in 3D with a novel method in rats. Tooth displacement was not constant at the different time periods, but conformed to a ‘V’ shape changing pattern. Although no OF was applied on the second molar, this tooth, as well as both molars at the no OF side, underwent significant displacement, in a similar pattern as that of the loaded first molar. The dynamic assessment of bone morphometry demonstrated that a higher OTM rate was concomitant with more alveolar bone loss, which provides instructive evidence for both clinical, translational and basic research in OTM.

## Supplementary Information

Supplementary Figure 1.

Supplementary Figure 2.

Supplementary Figure 3.

Supplementary Table 1.

Supplementary Table 2.

## Data Availability

All data generated or analyzed during this study are included in this published article and its supplementary information files.
