# Supplementary material for: Dynamic changes in tooth displacement and bone morphometry induced by orthodontic force
Source: Sci Rep. 2022 Aug 11;12:13672. doi: 10.1038/s41598-022-17412-8 (PMC9372182; doi:10.1038/s41598-022-17412-8)
Supplement: Supplementary file 1 — Supplementary Figure 1. [file 41598_2022_17412_MOESM1_ESM.docx]

**Supplementary Figure 1.**

| Arrival | 3 weeks before T0 | T0 | T1 | T2 | T3 | T4 |
| --- | --- | --- | --- | --- | --- | --- |
| - Adaptive period   of 2 weeks | - Mini-screw implantation - Healing time   of 3 weeks | - Micro-CT capture - Orthodontic force loading | - Micro-CT capture | - Micro-CT capture | - Micro-CT capture | - Micro-CT capture - Euthanasia |
