# Supplementary material for: Dynamic changes in tooth displacement and bone morphometry induced by orthodontic force
Source: Sci Rep. 2022 Aug 11;12:13672. doi: 10.1038/s41598-022-17412-8 (PMC9372182; doi:10.1038/s41598-022-17412-8)
Supplement: Supplementary file 2 — Supplementary Figure 2. [file 41598_2022_17412_MOESM2_ESM.docx]

**Supplementary Figure 2.**


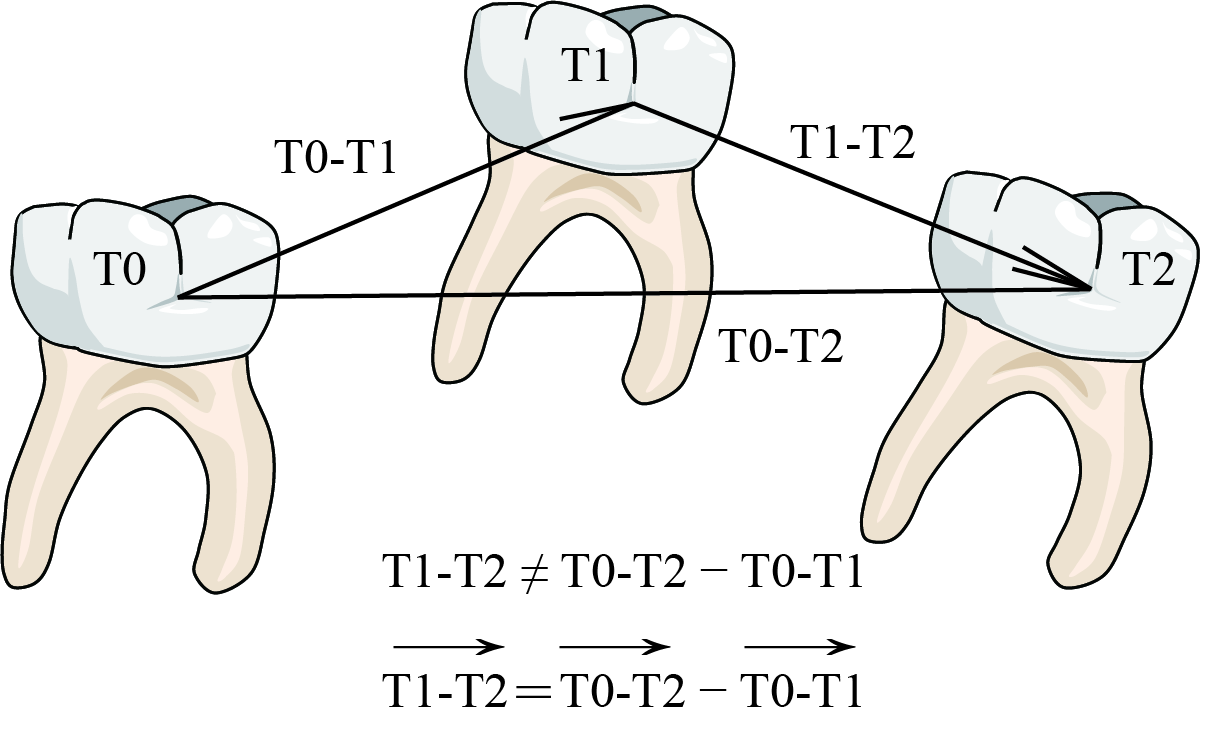


The true 3D displacement from T0 to T4 did not necessarily always happen in the same direction or plane. Thus, the evolution of OTM should be evaluated not simply by subtracting the OTM towards T0 of the adjacent periods (for example T0-T1 and T0-T2), but by directly measuring the OTM between each adjacent period (T1-T2).
