# Supplementary material for: Dynamic changes in tooth displacement and bone morphometry induced by orthodontic force
Source: Sci Rep. 2022 Aug 11;12:13672. doi: 10.1038/s41598-022-17412-8 (PMC9372182; doi:10.1038/s41598-022-17412-8)
Supplement: Supplementary file 3 — Supplementary Figure 3. [file 41598_2022_17412_MOESM3_ESM.docx]

**Supplementary Figure 3.**


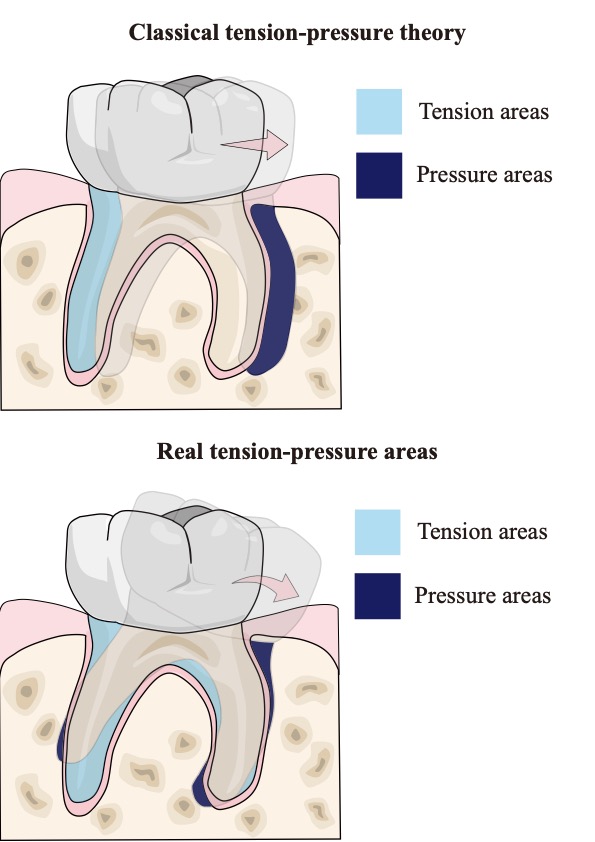


When the tooth moves mesially, classical tension-pressure theory claims that pressure and tension areas are the mesial and distal root areas. However, pure mesial body movement hardly exists, so the OTM is a mixture of body movement and angular movement. In this condition, the real tension-pressure areas are not distributed in two opposite sides, but actually dispersed, adjacent and widely connected in both mesial and distal sides.
