# Supplementary material for: Dynamic changes in tooth displacement and bone morphometry induced by orthodontic force
Source: Sci Rep. 2022 Aug 11;12:13672. doi: 10.1038/s41598-022-17412-8 (PMC9372182; doi:10.1038/s41598-022-17412-8)
Supplement: Supplementary file 4 — Supplementary Table 1. [file 41598_2022_17412_MOESM4_ESM.docx]

**Supplementary Table 1. OTM and OTM rate of the first and second molars in different time periods and hemi maxillae**

|  |  | First molar | | | | | | | |
| --- | --- | --- | --- | --- | --- | --- | --- | --- | --- |
|  |  | OTM | | | | | OTM rate | | |
|  |  | Occlusal | Apical | Angular | | Occlusal | | Apical | Angular |
| T0-T1 | No OF | 0.17 ± 0.07 | 0.15 ± 0.07 | 1.41 ± 0.99 | | 0.02 ± 0.01 | | 0.02 ± 0.01 | 0.14 ± 0.10 |
|  | OF | 0.44 ± 0.15 | 0.21 ± 0.08 | 6.49 ± 2.47 | | 0.04 ± 0.02 | | 0.02 ± 0.01 | 0.65 ± 0.25 |
| T0-T2 | No OF | 0.23 ± 0.09 | 0.26 ± 0.09 | 2.27 ± 1.43 | | 0.03 ± 0.01 | | 0.04 ± 0.01 | 0.32 ± 0.20 |
|  | OF | 0.68 ± 0.17 | 0.29 ± 0.12 | 9.65 ± 2.50 | | 0.10 ± 0.02 | | 0.04 ± 0.02 | 1.38 ± 0.36 |
| T0-T3 | No OF | 0.26 ± 0.10 | 0.29 ± 0.07 | 1.83 ± 1.73 | | 0.04 ± 0.01 | | 0.04 ± 0.01 | 0.26 ± 0.25 |
|  | OF | 1.00 ± 0.25 | 0.41 ± 0.19 | 14.30 ± 3.89 | | 0.14 ± 0.04 | | 0.06 ± 0.03 | 2.04 ± 0.56 |
| T0-T4 | No OF | 0.37 ± 0.19 | 0.37 ± 0.18 | 2.01 ± 1.52 | | 0.05 ± 0.03 | | 0.05 ± 0.03 | 0.29 ± 0.22 |
|  | OF | 1.59 ± 0.42 | 0.61 ± 0.27 | 23.90 ± 7.04 | | 0.23 ± 0.06 | | 0.09 ± 0.04 | 3.41 ± 1.01 |
| T1-T2 | No OF | 0.13 ± 0.05 | 0.14 ± 0.04 | 1.43 ± 1.23 | | 0.02 ± 0.01 | | 0.02 ± 0.01 | 0.20 ± 0.18 |
|  | OF | 0.29 ± 0.14 | 0.17 ± 0.06 | 4.62 ± 2.31 | | 0.04 ± 0.02 | | 0.02 ± 0.01 | 0.66 ± 0.33 |
| T2-T3 | No OF | 0.11 ± 0.06 | 0.12 ± 0.05 | 0.81 ± 0.33 | | 0.02 ± 0.01 | | 0.02 ± 0.01 | 0.12 ± 0.05 |
|  | OF | 0.39 ± 0.12 | 0.21 ± 0.10 | 5.10 ± 2.91 | | 0.06 ± 0.02 | | 0.03 ± 0.01 | 0.73 ± 0.42 |
| T3-T4 | No OF | 0.25 ± 0.12 | 0.25 ± 0.13 | 1.61 ± 1.11 | | 0.04 ± 0.02 | | 0.04 ± 0.02 | 0.23 ± 0.16 |
|  | OF | 0.65 ± 0.23 | 0.32 ± 0.15 | 10.07 ± 4.82 | | 0.09 ± 0.03 | | 0.05 ± 0.02 | 1.44 ± 0.69 |
|  |  | Second molar | | | | | | | |
|  |  | OTM | | | | OTM rate | | | |
|  |  | Occlusal | Apical | | Angular | Occlusal | | Apical | Angular |
| T0-T1 | No OF | 0.15 ± 0.08 | 0.16 ± 0.10 | | 1.42 ± 1.24 | 0.02 ± 0.01 | | 0.02 ± 0.01 | 0.14 ± 0.12 |
|  | OF | 0.23 ± 0.08 | 0.23 ± 0.08 | | 1.23 ± 0.59 | 0.02 ± 0.01 | | 0.02 ± 0.01 | 0.12 ± 0.06 |
| T0-T2 | No OF | 0.23 ± 0.07 | 0.26 ± 0.09 | | 1.69 ± 1.24 | 0.03 ± 0.01 | | 0.04 ± 0.01 | 0.24 ± 0.18 |
|  | OF | 0.35 ± 0.12 | 0.33 ± 0.10 | | 1.81 ± 0.70 | 0.05 ± 0.02 | | 0.05 ± 0.01 | 0.26 ± 0.10 |
| T0-T3 | No OF | 0.25 ± 0.08 | 0.30 ± 0.09 | | 1.88 ± 0.95 | 0.04 ± 0.01 | | 0.04 ± 0.01 | 0.27 ± 0.14 |
|  | OF | 0.42 ± 0.13 | 0.38 ± 0.13 | | 2.20 ± 0.89 | 0.06 ± 0.02 | | 0.05 ± 0.02 | 0.31 ± 0.13 |
| T0-T4 | No OF | 0.36 ± 0.15 | 0.40 ± 0.17 | | 2.48 ± 1.22 | 0.05 ± 0.02 | | 0.06 ± 0.02 | 0.35 ± 0.17 |
|  | OF | 0.59 ± 0.15 | 0.52 ± 0.16 | | 2.86 ± 1.46 | 0.08 ± 0.02 | | 0.07 ± 0.02 | 0.41 ± 0.21 |
| T1-T2 | No OF | 0.14 ± 0.07 | 0.14 ± 0.06 | | 1.12 ± 1.04 | 0.02 ± 0.01 | | 0.02 ± 0.01 | 0.16 ± 0.15 |
|  | OF | 0.19 ± 0.07 | 0.17 ± 0.07 | | 1.27 ± 0.58 | 0.03 ± 0.01 | | 0.02 ± 0.01 | 0.18 ± 0.08 |
| T2-T3 | No OF | 0.12 ± 0.06 | 0.11 ± 0.04 | | 1.18 ± 0.58 | 0.02 ± 0.01 | | 0.02 ± 0.01 | 0.17 ± 0.08 |
|  | OF | 0.17 ± 0.07 | 0.14 ± 0.07 | | 1.51 ± 0.68 | 0.02 ± 0.01 | | 0.02 ± 0.01 | 0.22 ± 0.10 |
| T3-T4 | No OF | 0.22 ± 0.12 | 0.22 ± 0.13 | | 1.04 ± 0.66 | 0.03 ± 0.02 | | 0.03 ± 0.02 | 0.15 ± 0.09 |
|  | OF | 0.27 ± 0.09 | 0.25 ± 0.12 | | 1.56 ± 0.99 | 0.04 ± 0.01 | | 0.04 ± 0.02 | 0.22 ± 0.14 |
