# Supplementary material for: Dynamic changes in tooth displacement and bone morphometry induced by orthodontic force
Source: Sci Rep. 2022 Aug 11;12:13672. doi: 10.1038/s41598-022-17412-8 (PMC9372182; doi:10.1038/s41598-022-17412-8)
Supplement: Supplementary file 5 — Supplementary Table 2. [file 41598_2022_17412_MOESM5_ESM.docx]

|  |  | First molar | | | | | |
| --- | --- | --- | --- | --- | --- | --- | --- |
|  |  | Occlusal | | Apical | | Angular | |
|  |  | 95% CI of diff. | *P* value | 95% CI of diff. | *P* value | 95% CI of diff. | *P* value |
| No OF^1^ | T0-T1 vs. T0-T2 | -0.089 to -0.051 | <0.01 * | -0.13 to -0.090 | <0.01 * | -1.9 to 0.18 | 0.14 |
|  | T0-T2 vs. T0-T3 | -0.054 to -0.0055 | <0.01 * | -0.049 to -0.0029 | 0.02 * | -0.76 to 1.6 | >0.99 |
|  | T0-T3 vs. T0-T4 | -0.15 to -0.066 | <0.01 * | -0.13 to -0.029 | 0.12 | -1.7 to 1.4 | >0.99 |
|  | T0-T1 vs. T1-T2 | 0.0095 to 0.066 | <0.01 * | -0.014 to 0.044 | >0.99 | -1.7 to 1.7 | >0.99 |
|  | T1-T2 vs. T2-T3 | -0.0057 to 0.033 | 0.3595 | 0.00042 to 0.041 | 0.04 * | -0.73 to 2.0 | >0.99 |
|  | T2-T3 vs. T3-T4 | -0.17 to -0.096 | <0.01 * | -0.17 to -0.093 | <0.01 * | -1.9 to 0.32 | 0.25 |
| OF^1^ | T0-T1 vs. T0-T2 | --0.28 to -0.20 | <0.01 * | -0.11 to -0.052 | <0.01 * | -4.9 to -1.4 | <0.01 * |
|  | T0-T2 vs. T0-T3 | -0.38 to -0.27 | <0.01 * | -0.16 to -0.075 | <0.01 * | -7.7 to -1.6 | <0.01 * |
|  | T0-T3 vs. T0-T4 | -0.69 to -0.51 | <0.01 * | -0.26 to -0.15 | <0.01 * | -13 to -5.7 | <0.01 * |
|  | T0-T1 vs. T1-T2 | 0.076 to 0.23 | <0.01 * | -0.0011 to 0.072 | 0.06 | -1.7 to 5.4 | 0.72 |
|  | T1-T2 vs. T2-T3 | -0.17 to -0.031 | <0.01 * | -0.073 to -0.0046 | 0.02 * | -4.2 to 3.3 | >0.99 |
|  | T2-T3 vs. T3-T4 | -0.34 to -0.18 | <0.01 * | -0.15 to -0.055 | <0.01 * | -9.9 to -0.089 | 0.05 * |
| No OF vs OF^2^ | T0-T1 | -0.33 to -0.24 | <0.01 * | -0.084 to -0.022 | <0.01 * | -7.5 to -2.6 | <0.01 * |
|  | T0-T2 | -0.50 to -0.40 | <0.01 * | -0.072 to 0.022 | 0.68 | -9.8 to -5.0 | <0.01 * |
|  | T0-T3 | -0.81 to -0.68 | <0.01 * | -0.18 to -0.056 | <0.01 * | -16 to -9.2 | <0.01 * |
|  | T0-T4 | -1.4 to -1.1 | <0.01 * | -0.33 to -0.15 | <0.01 * | -28 to -16 | <0.01 * |
|  | T1-T2 | -0.20 to -0.13 | <0.01 * | -0.055 to -0.013 | <0.01 * | -5.4 to -1.0 | <0.01 * |
|  | T2-T3 | -0.32 to -0.24 | <0.01 * | -0.12 to -0.064 | <0.01 * | -6.7 to -1.9 | <0.01 * |
|  | T3-T4 | -0.48 to -0.33 | <0.01 * | -0.11 to -0.021 | <0.01 * | -13 to -4.4 | <0.01 * |
|  |  | Second molar | | | | | |
|  |  | Occlusal | | Apical | | Angular | |
|  |  | 95% CI of diff. | *P* value | 95% CI of diff. | *P* value | 95% CI of diff. | *P* value |
| No OF^1^ | T0-T1 vs. T0-T2 | -0.094 to -0.060 | <0.01 * | -0.12 to -0.072 | <0.01 * | -1.8 to 1.2 | >0.99 |
|  | T0-T2 vs. T0-T3 | -0.047 to -0.0015 | 0.03 * | -0.062 to -0.018 | <0.01 * | -1.2 to 0.77 | >0.99 |
|  | T0-T3 vs. T0-T4 | -0.15 to -0.064 | <0.01 * | -0.16 to -0.043 | <0.01 * | -1.8 to 0.59 | 0.80 |
|  | T0-T1 vs. T1-T2 | -0.028 to 0.050 | >0.99 | -0.026 to 0.073 | >0.99 | -1.7 to 1.2 | >0.99 |
|  | T1-T2 vs. T2-T3 | -0.0035 to 0.044 | 0.14 | -0.00064 to 0.053 | 0.06 | -1.1 to 0.98 | >0.99 |
|  | T2-T3 vs. T3-T4 | -0.14 to -0.067 | <0.01 * | -0.16 to -0.052 | <0.01 * | -1.1 to 0.91 | >0.99 |
| OF^1^ | T0-T1 vs. T0-T2 | -0.14 to -0.10 | <0.01 * | -0.13 to -0.075 | <0.01 * | -1.4 to 0.22 | 0.24 |
|  | T0-T2 vs. T0-T3 | -0.091 to -0.048 | <0.01 * | -0.084 to -0.024 | <0.01 * | -1.3 to 0.47 | >0.99 |
|  | T0-T3 vs. T0-T4 | -0.20 to -0.13 | <0.01 * | -0.18 to -0.092 | <0.01 * | -1.7 to 0.35 | 0.36 |
|  | T0-T1 vs. T1-T2 | 0.011 to 0.069 | <0.01 * | 0.022 to 0.10 | <0.01 * | -1.1 to 0.70 | >0.99 |
|  | T1-T2 vs. T2-T3 | -0.0013 to 0.059 | <0.01 * | -0.0085 to 0.064 | 0.25 | -1.2 to 0.075 | 0.10 |
|  | T2-T3 vs. T3-T4 | -0.14 to -0.079 | <0.01 * | -0.16 to -0.049 | <0.01 * | -1.4 to 1.1 | >0.99 |
| No OF vs OF^2^ | T0-T1 | -0.097 to -0.068 | <0.01 * | -0.088 to -0.047 | <0.01 * | -1.2 to 1.6 | 0.99 |
|  | T0-T2 | -0.15 to -0.10 | <0.01 * | -0.10 to -0.039 | <0.01 * | -1.5 to 1.3 | >0.99 |
|  | T0-T3 | -0.21 to -0.14 | <0.01 * | -0.14 to -0.035 | <0.01 * | -1.4 to 0.81 | 0.89 |
|  | T0-T4 | -0.27 to -0.20 | <0.01 * | -0.15 to -0.079 | <0.01 * | -2.1 to 1.4 | 0.95 |
|  | T1-T2 | -0.085 to -0.023 | <0.01 * | -0.062 to 0.0029 | 0.09 | -0.80 to 1.3 | 0.95 |
|  | T2-T3 | -0.062 to -0.029 | <0.01 * | -0.047 to -0.0095 | <0.01 * | -1.1 to 0.56 | 0.82 |
|  | T3-T4 | -0.079 to -0.021 | <0.01 * | -0.069 to 0.013 | 0.29 | -2.2 to 1.5 | 0.97 |

**Supplementary Table 2. Evaluation of the effect of time and OF on first and second molar movement.**

Two-way repeated measures MANOVA and post-hoc Tukey’s test were used to compare the OTM between different time points (T0-T4) and between the OF and no OF side.

^1^ Pairwise comparisons by factor of time

^2^ Pairwise comparisons by factor of orthodontic force

* *P* < 0.05
